# A Prophylactic Clothes Philosophy

**Published:** 1896-02-08

**Authors:** 


The Hospital

An Institutional Journal of

The Medical Sciences and Hospital Administration, with

Vol. XIX.—No. 489.] AN EXTRA NURSING SUPPLEMENT. [Feb. 8, 1896.

### A Prophylactic Clothes Philosophy.

Carlyle, in "Sartor Resartus," gave to the world a "philosophy of clothes." Dr. W. F. Cleveland, in a much more modest form, has given to his medical brethren a clothes philosophy having a prophylactic purpose. The world wears clothes for ornament and dignity, argues Carlyle. Not so, says Dr. Cleveland. The world, in so far as it is a wise world, wears clothes for the regulation of bodily heat, and so for the preservation of health and life.

This business of the regulation of bodily heat is a kind of chemical speciality in the body. That is a thing which we, as medical men, often forget to think of. But let us think of it for a moment under Dr. Cleveland's guidance. In the first place there is a temperature peculiar to human organisms which is different from that of many other more or less similar organisms. From this it might be inferred, as is actually the fact, that there is a special mechanism in the body whose function it is to control the production of heat, and to so control it as to produce stability of amount. Science has demonstrated that there is in the brain a "heat centre," and that that centre, when the body is in normal health, works with so much precision and regularity as to keep the body generally at a practically unvarying temperature.

But though the human "heat centre" produces such admirable results when the whole body is in good health, that centre, like so many others, is very finely balanced, and is easily thrown out of good working order by many things which disturb the general health of the organism. A mere chill, for example, may so disarrange the mechanism of the heat centre as to bring about a rise of temperature in a very few hours. Now a rise of temperature always means a fall of health. It is here that Dr. Cleveland comes in with his prophylactic clothes philosophy. A summary of that philosophy may be put into a sentence. Regulate your clothing wisely, in accordance with the teaching of physiology and experience, and a whole class of bodily ills which arise from disturbance of the "heat centre" will be entirely prevented.

Is this another "mystery" of the physiological laboratory; a mystery which plain people can never understand, and which doctors smile at, but do not take into practical account? Most certainly not! Dr. Cleveland, like an old-fashioned but true man of science, makes his appeal direct to Nature. Does Nature provide for her sentient creatures any varieties of clothing in accordance with their changing environments of season, climate, or latitude, and their presumed varying necessities under these varying conditions? Most assuredly she does. A very striking example of this is found in a class of animals which have many of the peculiarities of land animals, and yet live in the sea. These are the whales, the dolphins, and other animals of the natural order Cetacea. Those warm-blooded creatures have to live in the cold waters of the seas, and some of them, as is well known, inhabit the coldest of all the seas. How do they manage to live at all? Nature provides them, as Dr. Cleveland reminds us, with an enormously thickened skin—a skin charged throughout with water-resisting oil. The "blubber" of the whale, which is its inner and oil-charged skin, is, in some parts of the body, many inches thick, and so effectually prevents the over-radiation of heat from the body within the skin to the cold water without it that the whale lives a comfortable and happy life in its freezing home. Not the whale only, but the duck and other water birds, as well as innumerable land animals, have special clothing provided by Nature for the prevention of undue radiation of heat and the preservation in a condition of healthy stability of the particular temperature of the body which is normal for each of them. The whole animal world, in short, bears testimony to the scientific and also to the common mind of the necessity for so clothing the body as to preserve it at a fixed temperature, and that the temperature of health.

From science to practice. Dr. Cleveland is very practical. Like a careful family physician, he is thinking of all the innumerable ills which his patients suffer from the damps and chills of an English climate, and in particular from the mists and fogs of London. Health in this country, he strenuously insists, is largely a question of clothing, of prophylactic, that is, preventive, clothing. As a matter of course the clothing must be of suitable material and of sufficient substance. But there is one principle more important than all the rest in clothing the body so as to secure true prophylaxis, or the prevention of such ills as arise from cold and damp. That principle is the wearing of a sufficiently thick and warm material *next the skin.* So important, indeed, is this principle that we think it might have been expanded at still greater length and fulness. Let us recur for a moment to the lower animals, and ask how their warm clothing is applied? The whale, for instance, wears his warm coat of blubber, not on his skin, but inside it. Similarly the hare and the fox, and other animals which grow winter coats, have them not merely close to the skin, but actually growing from it. The *sine quâ non* then of clothing oneself prophylactically against chills and damps and all the vicissitudes of a fickle climate is to wear clothing which is at once non-conducting and sufficiently thick, and clothing which is so accurately fitted to the figure throughout that it will exclude the outer air from the surface of the body.

